# Geochemically induced shifts in catabolic energy yields explain past ecological changes of diffuse vents in the East Pacific Rise 9°50'N area

**DOI:** 10.1186/1467-4866-13-2

**Published:** 2012-01-27

**Authors:** Michael Hentscher, Wolfgang Bach

**Affiliations:** 1 Department of Geosciences, University of Bremen, Klagenfurter Straße, 28359 Bremen, Germany

## Abstract

The East Pacific Rise (EPR) at 9°50'N hosts a hydrothermal vent field (Bio9) where the change in fluid chemistry is believed to have caused the demise of a tubeworm colony. We test this hypothesis and expand on it by providing a thermodynamic perspective in calculating free energies for a range of catabolic reactions from published compositional data. The energy calculations show that there was excess H_2_S in the fluids and that oxygen was the limiting reactant from 1991 to 1997. Energy levels are generally high, although they declined in that time span. In 1997, sulfide availability decreased substantially and H_2_S was the limiting reactant. Energy availability dropped by a factor of 10 to 20 from what it had been between 1991 and 1995. The perishing of the tubeworm colonies began in 1995 and coincided with the timing of energy decrease for sulfide oxidizers. In the same time interval, energy availability for iron oxidizers increased by a factor of 6 to 8, and, in 1997, there was 25 times more energy per transferred electron in iron oxidation than in sulfide oxidation. This change coincides with a massive spread of red staining (putative colonization by Fe-oxidizing bacteria) between 1995 and 1997.

For a different cluster of vents from the EPR 9°50'N area (Tube Worm Pillar), thermodynamic modeling is used to examine changes in subseafloor catabolic metabolism between 1992 and 2000. These reactions are deduced from deviations in diffuse fluid compositions from conservative behavior of redox-sensitive species. We show that hydrogen is significantly reduced relative to values expected from conservative mixing. While H_2 _concentrations of the hydrothermal endmember fluids were constant between 1992 and 1995, the affinities for hydrogenotrophic reactions in the diffuse fluids decreased by a factor of 15 and then remained constant between 1995 and 2000. Previously, these fluids have been shown to support subseafloor methanogenesis. Our calculation results corroborate these findings and indicate that the 1992-1995 period was one of active growth of hydrogenotrophic communities, while the system was more or less at steady state between 1995 and 2000.

## Introduction

**Figure 1:**
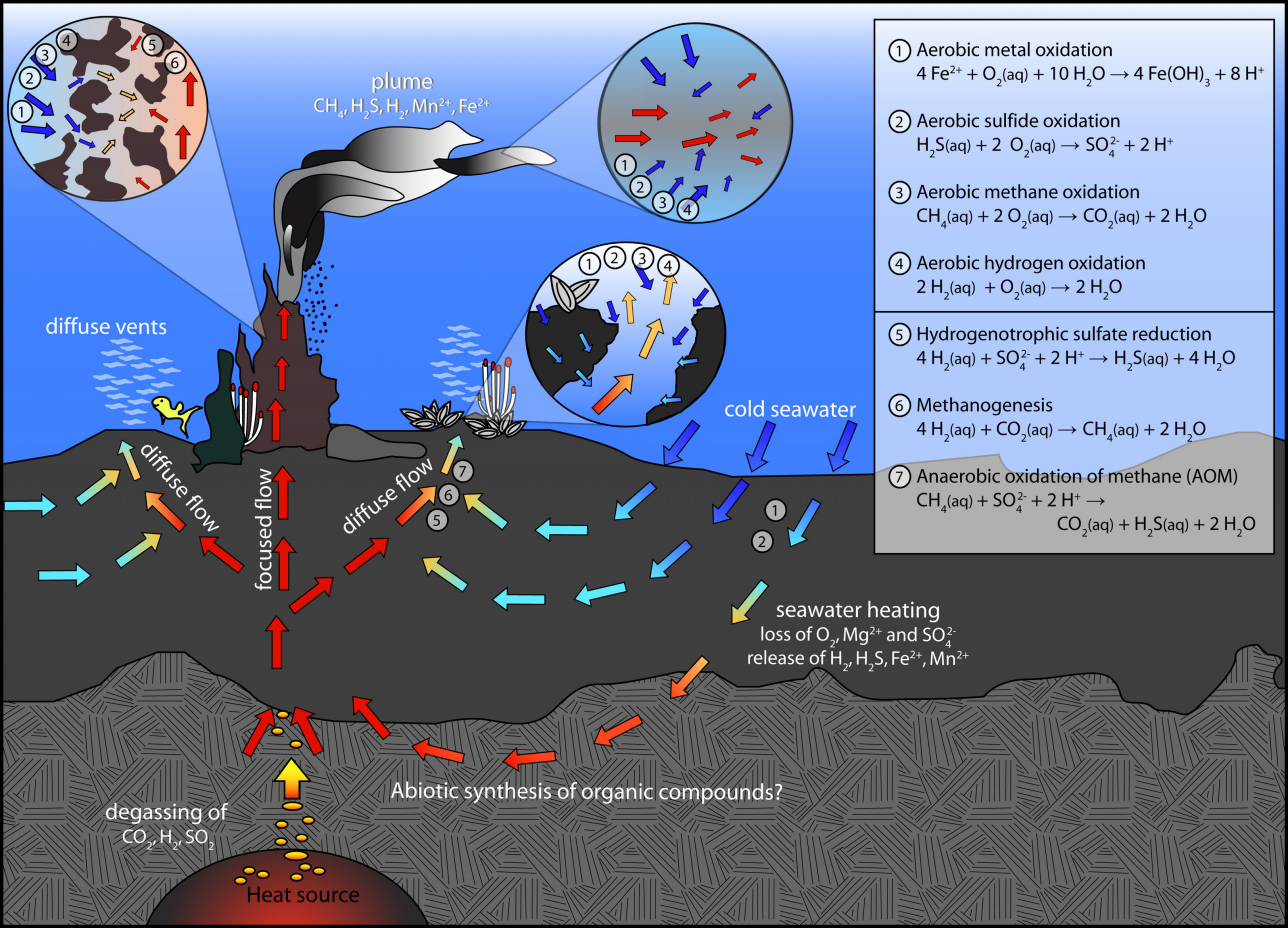
**Sketch of idealized fluid flow within a hydrothermal system and potential catabolic reactions in different environments (chimney wall, plume, recharge zone, and subseafloor mixing zone)**. Upwelling hot, reducing hydrothermal fluids mix with entrained cold, oxygenated seawater in subseafloor mixing zones. For these zones, the affinities of the catabolic reactions provided in the inset are examined in this paper.

Microorganisms have the ability to gain energy for their metabolism by promoting a large range of redox reactions. Well-known energy sources are for example aerobic oxidation of methane or hydrogen sulfide, methanogenesis, fermentation, and sulfate reduction under anaerobic conditions [1]. In habitats like hydrothermal systems or mines, lacking sunlight and organic carbon sources, the primary production depends on electron donors that are released by water-rock reactions. High-temperature (> 400°C) processes of water-rock interaction determine the composition of seawater-derived hydrothermal fluids that are equilibrated with rocks at depths as much as several kilometers (Figure 1). Upon upwelling, these fluids cool (conductively and/or adiabatically) and mix with cold seawater to varying extents. High temperature fluids, venting focused via black smoker chimneys, often show little evidence for subseafloor mixing and are typically used as "hydrothermal endmember" compositions. Commonly, sites of diffuse venting are developed around the black smokers, and the temperature-composition relations of the fluids issuing through the seafloor there indicate that the diffuse fluids formed by subseafloor cooling and mixing of hot hydrothermal fluids with cold seawater. The seafloor underneath these diffuse vent sites is a particularly favorable environment for a variety of chemosynthetic microorganisms in terms of suitable temperature and large energy availability (Figure 1). The composition of the upwelling hydrothermal fluids in these diffuse vent sites imposes a major control on the metabolic diversity in the colonizing ecosystem. Because of this tight relation between vent ecosystem and fluid compositions, chemical changes in the fluid may directly influence the ecosystem.

Thermodynamic calculations based on geochemical compositions of waters in these habitats provide insights into the energy availability and can determine possible reactions that can support primary production in these systems [2-5]. Tight relations between the availability of geochemical energy and microbial processes have been demonstrated for a variety of submarine hydrothermal environments, including chimney walls, diffuse fluids, and vent mussels [6-10].

In this study we use geochemical data from two hydrothermally active vents in the East Pacific Rise 9°50'N area to show that thermodynamic modeling can help interpret the microbial metabolism in such systems. For the first area, our calculations provide clues to the biological evolution of a vent site influenced by dynamic changes in fluid chemistry and, consequently, catabolic energy. The other case shows that microbial processes in the subseafloor may be deciphered by determining and comparing free energies of reactions for catabolic reactions of hypothetical fluids derived from conservative mixing of seawater and hydrothermal fluid with the diffuse fluid actually sampled.

## Methods

### Calculation of affinity

Free energy for catabolic reactions is available only if the system is out of geochemical equilibrium. Disequilibrium prevails when the properties of the system change at rates faster than the rates at which the thermodynamically favored reactions proceed. The abiotic rates of many redox reactions are sluggish, in particular at temperatures conducive of life (< 120°C)[5]. Microbes use enzymes to catalyze these redox reactions and harness the free energy by controlling electron transfers and converting a sizable fraction of the catabolic energy in ATP production for their anabolic metabolism [11]. The maximum quantity of free energy that microorganisms can catabolize (Δ_r_G) is given by the Gibbs energy at a reference state (Δ_r_G° = -RTlnK_r_) representing the intensive parameters (P, T) and an extensive term (RTlnQ) that captures the compositions of the vent solutions (equation 1):

$$\Delta_{\text{r}}\text{G}=-\text{RTln}\left({\text{K}}_{\text{r}}\right)+\text{RTln}\left({\text{Q}}_{\text{r}}\right)$$

where R is the universal gas constant and T the temperature in Kelvin. K_r _is the calculated equilibrium for the temperature and pressure of interest, and Q_r _expresses the activities of species participating a specific reaction. Q_r _is evaluated through equation (2):

$${\text{Q}}_{\text{r}}=\underset{\text{i}}{\prod }{{\text{a}}_{\text{i}}}^{{\text{v}}_{\text{ir}}}$$

where a_i _represents the activity of the chemical species in the reaction, v_ir _denotes the stoichiometric coefficient for the i*th *chemical species in the reaction, which is positive for products and negative for reactants. If Δ_r_G for a reaction is negative, then the reaction should proceed from left to right; if it is greater than zero, the reaction will proceed in the opposite direction. By convention a negative sign indicates that the reaction should take place spontaneously and energy can be gained by microbes catalyzing this reaction.

Commonly, affinity is used instead of Δ_r_G for a reaction [12]. Affinities express the change of the Gibbs energy with reaction progress (ξ) (equation 3):

$${\text{A}}_{\text{r}}=\;-{\left(\partial \Delta_{\text{r}}\text{G}/\partial \xi \right)}_{\text{P},\text{T}}$$

It follows that the reaction is favorable if the affinity is positive. Combining equations 1 and 3, the affinity can be evaluated through equation (4):

$${\text{A}}_{\text{r}}=\text{ RT ln}\left({\text{K}}_{\text{r}}/{\text{Q}}_{\text{r}}\right)$$

This relation demonstrates that, if K_r _> Q_r_, then A_r _> 0 and the reaction may proceed while free energy is released [12].

Two types of computations were employed in this study: (1) calculation of concentrations and activities of dissolved species in diffuse fluids, and (2) calculation of affinities of potential catabolic reactions in these fluids. These calculations were conducted for actual diffuse fluids sampled and analyzed by Von Damm and Lilley [13] and for hypothetical mixtures of endmember vent fluids [14] and ambient seawater. It is assumed here that the diffuse fluids form by subseafloor mixing of ascending hydrothermal fluids with seawater. The endmember hydrothermal fluid composition is taken from black smoker vent fluids issuing within a few meters of the diffuse vent site [13,14]. The percentage of hydrothermal fluid is estimated using a simple mass balance for silica:

$$\begin{array}{c} \text{Hydrothermal Fluid \% }=\\ \text{100}∙\frac{{\text{C}}_{\text{Si}{\text{O}}_{\text{2}}\text{(aq)diffuse fluid}}\text{ - }{\text{C}}_{\text{Si}{\text{O}}_{\text{2}}\text{(aq), sw}}}{{\text{C}}_{\text{Si}{\text{O}}_{\text{2}}\text{(aq)hydrothermal fluid}}\text{ - }{\text{C}}_{\text{Si}{\text{O}}_{\text{2}}\text{(aq),sw}}} \end{array}$$

Silica is known to precipitate slowly at low temperatures from mildly acidic fluids [15] and can be assumed to behave conservatively at the time scales of fluid mixing [16].

Geochemist Workbench^® ^(GWB) was used to conduct the thermodynamic calculations [17]. A Log K_r _database was created, covering temperatures from 0 to 350°C at a pressure of 25 MPa, using SUPCRT92 [18] and the thermodynamic database OBIGT [19], and including all speciation reactions in an aqueous system with Na, Ca, Mg, Fe, Sr, K, SiO_2_, Cl, sulfate, sulfide, oxygen, hydrogen and carbon dioxide. Likewise, equilibrium constants for the following catabolic reactions were calculated.

Aerobic sulfide oxidation

(1) $${\text{H}}_{2}\text{S}\left(\text{aq}\right)+2{\text{O}}_{2}\left(\text{aq}\right)\to {\text{SO}}_{4}^{2-}+2{\text{H}}^{+}$$

Aerobic methane oxidation

(2) $$\text{C}{\text{H}}_{4}\left(\text{aq}\right)+2{\text{O}}_{2}\left(\text{aq}\right)\to \text{C}{\text{O}}_{2}\left(\text{aq}\right)+2{\text{H}}_{2}\text{O}$$

Aerobic iron oxidation

(3) $$\text{F}{\text{e}}^{2+}+\;1/4\;{\text{O}}_{2}\left(\text{aq}\right)+2.5{\text{H}}_{2}\text{O}\to \text{Fe}{\left(\text{OH}\right)}_{3}+\;2{\text{H}}^{+}$$

Aerobic hydrogen oxidation

(4) $$2{\text{H}}_{2}\left(\text{aq}\right)+{\text{O}}_{2}\left(\text{aq}\right)\to 2{\text{H}}_{2}\text{O}$$

Hydrogenotrophic sulfate reduction

(5) $$4{\text{H}}_{2}\left(\text{aq}\right)+{\text{SO}}_{4}^{2-}+2{\text{H}}^{+}\to {\text{H}}_{2}\text{S}\left(\text{aq}\right)+4{\text{H}}_{2}\text{O}$$

Hydrogenotrophic methanogenesis

(6) $$4{\text{H}}_{2}\left(\text{aq}\right)+\text{C}{\text{O}}_{2}\left(\text{aq}\right)\to \text{C}{\text{H}}_{4}\left(\text{aq}\right)+2{\text{H}}_{2}\text{O}$$

Anaerobic oxidation of methane

(7) $$\text{C}{\text{H}}_{4}\left(\text{aq}\right)+{\text{SO}}_{4}^{2-}+2{\text{H}}^{+}\to {\text{H}}_{2}\text{S}\left(\text{aq}\right)+\text{C}{\text{O}}_{2}\left(\text{aq}\right)+2{\text{H}}_{2}\text{O}$$

Published compositions of endmember vent fluids [13,14] issuing from black smoker chimneys in proximity (few meters) to the diffuse vent site were used in the calculations of affinities for these reactions (Table 1). In determining Q_r _(equation2), the extended Debye-Hückel equation was used to calculate activity coefficients with extended parameters and hard core diameters for each species from Wolery and Jove-Colon [20]. Dissolved neutral species were assigned an activity coefficient of one, except non-polar species for which CO_2 _activity coefficients were used [21]. Reported pH values of hydrothermal vents (measured at 25°C) were used in determining the *in situ *pH (Table 1) by re-speciating the fluids at the temperatures of venting [22]. The percentage of hydrothermal endmember fluid in the diffuse fluids derived from the silica mass balance was used to calculate idealized mixed fluids, assuming conservative behavior of all elements. These hypothetical fluids were also speciated and compared with actual compositions of diffuse fluids in terms of concentrations and affinities (Tables 1 and 2). Deviations from conservative behavior in the diffuse fluids indicate that removal or release processes take place in the subseafloor mixing zones in which the diffuse fluids are formed. In the calculations, the activities of species in the hypothetical diffuse fluids (ideal conservative mixing) were determined in a batch mixing model simulating titration of hot hydrothermal endmember fluid into cold seawater and tracking the chemical speciation changes in the mixed fluid. In these calculations, redox reactions were suppressed, while kinetically fast reactions like protonation of bases, dissociation of acids, and complex formation are allowed to take place spontaneously. Redox reactions were suppressed, because these reactions are not expected to proceed at the low temperatures of the diffuse fluids and on the short time scales of the mixing process [23]. This procedure has the advantage that disequilibria formed during mixing can be determined and the affinities of selected redox reactions may be calculated. GWB also allows suppressing the Knallgas reaction, so elevated concentrations of both O_2 _and H_2 _in the mixed fluids could be accounted for [9].

**Table 1:** Compositions of discrete and diffuse vent fluids

		Temperature (°C)	pH _(25°C) _(1)	in situ pH	Mg^2+ ^mM	Na^+ ^mM (1)	Cl^+ ^mM	SiO_2 _(aq) mM	H_2 _(aq) μM	H_2_S (aq) mM	Fe^2+ ^μM	CH_4 _(aq) μM	CO_2 _(aq) mM	O_2 _(aq) μM (5)	SO_4_^2- ^mM (1, 6)	% hydrothermal fluid (4)
	**Seawater**	1.8	7.8	8.1	52.2	464	540	0.155	0	0	0	0	2.30	100	28.2	0

**Northern Area**																

April 1991	hot endmember	368	2.6	3.2	0	139	154	9.90	3030	23.2	2190	172	44.8	-	0	100
	diffuse, measured	22	-	-	49.7	-	530	0.88	0.36	0.90	151	0.070	5.90	-	-	7.39
	diffuse, calculated	22	5.7	5.7	48.3	440	511	0.88	225	1.72	162	12.8	5.46	92.6	26.1	7.39

December 1993	hot endmember	365	3.6	5.7	0	188	212	11.3	700	7.30	1060	1000	204	-	0.18	100
	diffuse, measured	30.9	-	-	49.6	-	522	0.57	0.33	0.28	24.2	5.80	9.57	-	-	3.68
	diffuse, calculated	30.9	5.5	5.5	50.3	454	528	0.57	25.9	0.27	39.3	37.1	9.77	96.3	27.2	3.68

March 1994	hot endmember	363	3.5	5.1	0	222	249	12.6	680	8.50	1430	112	187	-	0.035	100
	diffuse, measured	29.9	-	-	50.4	-	525	0.79	1.90	0.27	25.0	6.58	9.57	-	-	5.06
	diffuse, calculated	29.9	5.4	5.4	49.6	452	525	0.79	34.5	431	72.5	5.68	11.7	94.9	26.8	5.06

October 1994	hot endmember	364	3.2	3.8	0	279	325	14.1	530	6.20	2730	86.0	146	-	1.55	100
	diffuse, measured	32.3	-	-	48.1	-	524	0.92	6.79	0.11	69.9	4.83	8.54	-	-	5.45
	diffuse, calculated	32.3	5.5	5.4	49.3	454	528	0.92	29.2	0.34	150	4.74	10.2	94.5	26.7	5.45

November 1995	hot endmember	366	3.0	3.9	0	391	466	14.8	360	6.70	6030	84.0	139	-	0	100
	diffuse, measured	33.3	-	-	46.3	-	550	1.14	2.50	0.19	277	5.37	9.01	-	-	6.69
	diffuse, calculated	33.3	5.4	5.3	48.7	459	535	1.14	24.2	0.45	406	5.65	11.5	93.3	26.3	6.69

November 1997	hot endmember	373	3.1	4.1	0	342	400	13.4	330	8.60	6640	95.0	117	-	0	100
	diffuse, measured	27.2	-	-	50.5	-	534	0.72	0.75	0.003	170	2.52	4.70	-	-	4.23
	diffuse, calculated	27.2	5.7	5.7	50.0	459	534	0.72	14.1	0.37	284	4.07	7.21	95.7	27.0	4.23

**Southern Area**																

April 1991	diffuse, measured	55	-	-	46.7	-	504	1.95	2.17	8.46	2.40	505	10.1	-	-	-

February-March	hot endmember	160	3 (2)	3.7	0	126 (3)	136	12.7	6460	20.7	4080	213	31.0	-	-	100
1992	diffuse, measured	23.3	-	-	48.2	-	529	0.69	14.9	0.66	2.00	94.0	4.57	-	-	4.28
	diffuse, calculated	23.3	6.2	6.2	50.0	479	523	0.69	277	0.89	175	9.12	3.53	95.7	27.0	4.28

December 1993	diffuse, measured	15.4	-	-	50.5	-	532	0.23	0.13	0.001	9.20	2.87	2.70	-	-	-

March 1994	hot endmember	20.5	-	-	0	-	269	5.57	8910	11.2	200	748	118	-	-	100

October 1994	hot endmember	351	3 (2)		0	229 (3)	235	12.5	8390	14.3	1590	116	104	-	-	100
	diffuse, measured	20.4	-	-	49.7	-	526	0.66	6.75	0.21	11.5	15.6	4.96	-	-	4.05
	diffuse, calculated	20.4	-	5.8	50.1	484	528	0.66	343	0.58	65.0	4.74	6.46	95.9	27.0	4.05

November 1995	hot endmember	341	3 (2)	3.7	0	297 (3)	301	13.8	4930	14.0	1550	106	95.7	-	-	100
	diffuse, measured	24.7	-	-	50.3	-	531	0.85	0.75	0.53	9.90	15.9	5.80	-	-	5.06
	diffuse, calculated	24.7	5.7	5.7	49.5	484	528	0.85	250	0.71	78.7	5.38	7.04	94.9	26.8	5.06

November 1997	hot endmember	307	3 (2)	3.4	0	326 (3)	330	16.1	3550	11.3	742	121	81.9	-	-	100
	diffuse, measured	18.2	-	-	51.5	-	530	0.57	0.67	0.24	27.0	4.09	3.84	-	-	2.57
	diffuse, calculated	18.2	6.0	6.1	50.9	490	535	0.57	91.3	0.29	19.1	3.11	4.35	97.4	27.5	2.57

April 2000	hot endmember	279	3 (2)	3.3	0	355 (3)	359	16.8	2700	11.2	517	108	77.0	-	-	100
	diffuse, measured	11.9	-	-	50.4	-	529	0.23	0.15	0.055	23.0	0.47	2.57	-	-	0.42
	diffuse, calculated	11.9	7.0	6.9	52.0	494	539	0.23	12.5	0.051	2.39	0.50	2.65	99.5	28.1	0.42

Fluid data for high temperature fluids and measured diffuse fluids are from Von Damm and Lilley, 2004 [13]

Northern Area: Hot vent fluids are from Bio9 and Bio9' and the associated diffuse fluids from BM9Rifia, BM91o and BM12

Southern Area: High temperature fluids are from Tube Worm Pillar (TWP) and the diffuse fluids from Y vent at the base of TWP

(1) pH, sodium and sulfate concentrations of vent fluids are from Von Damm [14], unless otherwise indicated

(2) pH was not measured but is approximated by comparison with similar vent fluids from Von Damm [14]

(3) Na^+ ^calculated by charge balance

(4) Calculated assuming conservative behavior of SiO_2_(aq)

(5) O_2_(aq) in diffuse fluids was calculated from conservative mixing, assuming 100 μMol O_2 _for pacific bottom seawater [30]

(6) When sulfate concentrations are not reported, it is assumed to be zero in the endmember vent fluids

**Table 2:** Affinities for different catabolic reactions in kJ and normalized affinities in J per e^- ^and Kg Vent-fluid at the Southern Area (TWP)

Southern Area	5 Hydrogenotrophic sulfate reduction		6 Hydrogenotrophic methanogenesis		7 Anaerobe oxidation of methane	
	**Measured**	**Predicted**	**Measured**	**Predicted**	**Measured**	**Predicted**

	kJ		kJ		kJ	

February-March 1992	128.6	156.7	94.1	128.0	34.5	28.7
October 1994	129.3	165.1	93.4	135.3	35.9	29.8
November 1995	104.3	161.2	70.1	130.8	34.2	30.4
November 1997	103.9	151.1	73.8	122.5	30.1	28.7
April 2000	88.4	130.4	63.9	105.7	24.7	24.5

	J per e^- ^and Kg vent fluid		J per e^- ^and Kg vent fluid		J per e^- ^and Kg vent fluid	

February-March 1992	1.34	30.28	0.98	24.73	9.07	0.73
October 1994	0.64	41.50	0.46	34.01	1.64	0.41
November 1995	0.05	23.57	0.03	19.13	1.27	0.38
November 1997	0.08	16.34	0.06	13.24	0.58	0.42
April 2000	0.09	10.95	0.06	8.88	0.31	0.33

The precipitation of minerals was also suppressed. The thermodynamically stable Fe-minerals in the diffuse fluids are hematite and pyrite. If these phases were allowed to precipitate, Fe concentrations would drop to extremely low values in the hypothetical mixed fluids. The measured diffuse fluids have Fe concentrations that are many orders of magnitude higher than values corresponding to pyrite and hematite solubility. They are hence strongly oversaturated with respect to pyrite and hematite and indicate that precipitation of these minerals was largely inhibited. The concentrations of Fe and H_2_S calculated for the hypothetical mixed fluids represent maximum values.

The affinities calculated (Table 2) represent the maximum energy content for the different catabolic reactions, disregarding the fact that limiting electron donors and acceptors, which appear in several reactions, can still only be used once within the ecosystem [24]. Moreover, comparisons of the raw affinities do not reflect differences in the numbers of electrons transferred in these reactions. This is problematic, because a given quantity of proton motive force driving chemiosmosis is generated by a set number of electrons transferred. The fact that the reactions considered have between one and eight electron transferred therefore skews a comparison of the affinities of different reactions. We hence report the affinities in values per electron transferred (Table 2). Furthermore, the energy flux into the system is controlled by the concentration of the limiting reactant in the upwelling fluid. To examine these combined effects on energy availability, we normalized affinity to kg vent fluid by multiplying the energy with the concentration of the limited reactant, and divided by the fraction of endmember vent fluid in the mix [24]. These normalized affinities provide us with a meaningful parameter for assessing the fluxes of energy for different catabolic reactions into a system. While affinities expressed in both notations are reported in Table 2, the following discussion will primarily use normalized affinity, i.e., energy flux.

## Results and Discussion

### Case studies

The sample locations are situated in the axial summit caldera of the fast spreading (11 cm/yr full rate) ridge EPR at 9°50'N at a water depth of 2500 meters. In both case studies we use data from time series studies conducted in the 1990s. Venting temperatures are ≤55°C, and fluids issue from cracks in the seafloor or from lava pillars that are fissured near the base. Compositions of vent fluids from the two sites are reported in Von Damm and Lilley [13] and Von Damm [11]. These publications also present a detailed description of the geological setting and vent field characteristics, so we here highlight only the key features of these localities.

The northern area is characterized by the high temperature vents Bio9 and Bio9' and the associated diffuse flow sites BM9Riftia (BM9R), BM91o and BM12. The data set for this system (hereinafter referred to as Bio9 area) is the most detailed, because the site was the target of long-term measurements of fluid composition and temperature [25] and seismic activity [26]. Sohn et al. [26] documented a seismic swarm in 1995 in this area, followed by a temperature increase in the Bio9 vent with a delay of a few days [25]. Temperatures of the diffuse fluid samples range between 22.0 and 33.3°C (Table 1).

The southern area (Tube Worm Pillar, TWP) features high temperature venting through an 11-m high sulfide structure on top of a lava pillar. Discrete venting of 351°C fluid is restricted to the top of the chimney, while leakage of diffuse fluids is observed from around the base of the chimney. Eponymous for the site name, a large tubeworm colony inhabits the area of diffuse venting. The associated diffuse fluid samples were retrieved from Y vent, an adjacent broken-off lava pillar that issued fluids of temperatures between 20 and 25°C in 1992-1995, dropping to 18°C in 1997 and finally to 12°C in 2000.

### Case study 1 - Bio9 area

Shank et al. [27] studied the change in the vent community during the time period from 1991 to 1995 at the vents in the Bio9 area. These authors report of a magmatic event in 1991, followed by venting of fluids high in hydrogen sulfide. These conditions boosted the establishment of a strong population of the tubeworm *Riftia*. During the following cruises in 1994, Shank et al. [27] observed the development of rusty spots that appeared within the *Riftia *colonies. In 1995, the rusty spots had spread and covered large areas of the *Riftia *population. In 1997, the *Riftia *population had broken down largely, while the rust had extended to cover much of the *Riftia *patch [13].

**Figure 2:**
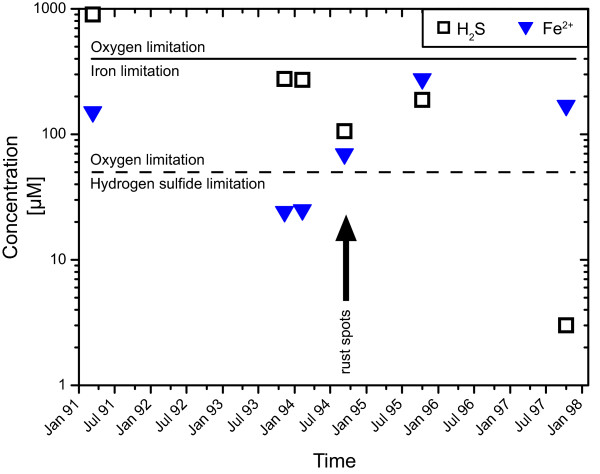
**Temporal changes in concentrations of dissolved iron and hydrogen sulfide in diffuse fluids from the Bio9 area**. The increase in iron coincides with the appearance of rusty spots in the tubeworm colony (black arrow). The two horizontal lines represent the maximum concentrations of sulfide (dashed) and iron (continuous) that can be oxidized by seawater with an oxygen concentration of 100 μM. Iron is the limiting reactant over the whole time period, in contrast to sulfide, which is oxygen limited, except for the conditions in November 97.

The temporal evolution of the fluid compositions in that time span reveals a decrease in hydrogen sulfide concentrations over the entire period after 1992 with a slight increase in November of 1995 (Figure 2). Before March of 1994, soluble iron follows the hydrogen sulfide concentration; afterwards the iron content increased and reached maximum concentrations during November of 1995. In November of 1997, the Fe concentration had dropped slightly, but was still much higher than during the beginning of the time series.

It has been suggested that the biological development of this area depends on the bioavailability of iron and H_2_S [13]. This interpretation is plausible, because *Riftia *live in symbiosis with sulfide oxidizing bacteria [28] and depend on the energy associated with sulfide oxidation. Also, Fe-oxidizing bacteria oxidize Fe^2+ ^in the fluids to ferric hydroxide [29]. So the "rust" in the study area is an indicator that these microorganisms are thriving.

We determined the affinities for both catabolic pathways for the time period of critical geochemical and ecological changes (1991-1997) to improve the understanding of the biological evolution of the vent ecosystems. The calculations make use of the measured concentrations of iron, H_2_S and oxygen. Unfortunately concentrations of oxygen and the pH for diffuse fluids are not available; therefore, these values are estimated from conservative mixing. Calculated pH values for the fluids show a narrow range of 5.3 to 5.7; likewise, small variations are predicted for oxygen concentrations (92 - 96 μM). Both pH and O_2 _concentrations reflect the large fraction of seawater calculated from the silica mass balance. Depending on the mixing ratio of vent fluid and seawater, either one of the electron donors (Fe^2+^, H_2_S) or oxygen is the limiting reactant determining the amount of energy available per unit vent fluid based (Figure 2). Figure 2 shows the upper limit for iron and H_2_S oxidation based on an oxygen concentration of the East Pacific bottom seawater of circa 100 μM oxygen [30]. For H_2_S oxidation, O_2 _is the limiting reactant, while Fe-oxidation is limited by the availability of iron. An exception is the fluid sampled last in the time series; it exhibits exceptionally low sulfide concentrations and H_2_S is the compound limiting energy availability.

**Figure 3:**
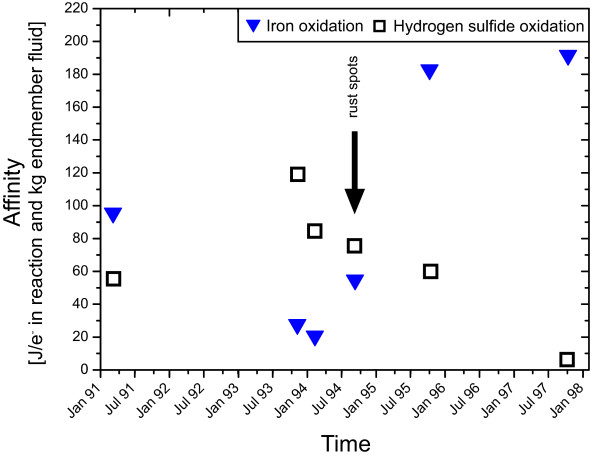
**Normalized affinities for the oxidation of Fe^2+ ^and H_2_S in diffuse fluids from the Bio9 area during the period from April 1991 until November 1997**. The generally high affinities for iron and hydrogen sulfide oxidation support life catabolizing these reactions. In October of 1994, affinities for both reactions are high, so that tubeworms (H_2_S-oxidizers) and iron oxidizing microorganism (rusty staining) can grow simultaneously. The demise of the *Riftia *population in November of 1997 coincides with a sudden drop in the affinity of H_2_S oxidation.

Figure 3 illustrates the normalized affinities for both reactions. It shows the consequence of limitation; sulfide oxidation has the highest affinity when the fraction of vent fluid in the mixture is lowest (Table 1), because then oxygen contents are greatest. In contrast, the normalized affinities for iron oxidation more closely mirror the iron concentration in the fluid. But affinities are also dependent on the vent fluid fraction, as increased pH favors ferric hydroxide precipitation from the mixed fluids.

The dynamic changes in the normalized affinities of sulfide and iron oxidation (Figure 3 and Additional File 1) can fully explain the ecological changes within the system. The incipient occurrence of rusty staining in November of 1994 correlates with an increased normalized affinity of iron oxidation, while the normalized affinity for sulfide oxidation remains at the same level. In November of 1995 a further increase in iron concentration in the fluid explains the continued spreading of the iron oxide staining. Tied to this change, the normalized affinity for Fe-oxidation almost quadrupled. The normalized affinity for hydrogen sulfide oxidation was only slightly decreased relative to 1994, which explains why the tubeworm colonies were still thriving, despite the increased development of rusty staining. Apparently, both metabolic pathways were favorable and were being exploited at that stage of system evolution. After 1995, the normalized affinity for hydrogen sulfide oxidation dropped as a consequence of the strongly decreased sulfide concentration in the fluid. Because of this drop in the affinity of sulfide oxidation, the tubeworm population, relying on favorable energetics for H_2_S oxidation, collapsed. Unlike sulfide oxidation, the normalized affinity for iron oxidation remains high, so organisms with the ability to gain energy from iron oxidation can still thrive. Since both reactions depend on oxygen, the reactions are in competition for that electron acceptor and the calculated affinities (Figure 3) are the predicted maxima.

The thermodynamic calculations presented here validate the interpretation by Von Damm and Lilley [13] and confirm that the ecological changes are driven by changes in fluid composition.

### Case study 2 - Tube Worm Pillar (TWP)

The fluid compositions of the diffuse fluids issuing in TWP area have been proposed to reveal insights in the redox reactions in the subseafloor [13,31]. Increased methane concentrations in the diffuse fluids led Von Damm and Lilley [13] to propose that hydrogenotrophic methanogenesis takes place in the subseafloor. Proskurowski et al. [31] could confirm this interpretation through carbon stable isotope measurements of methane and CO_2 _demonstrating that the carbon isotope ratios are consistent with active microbial carbon cycling in this area.

**Figure 4:**
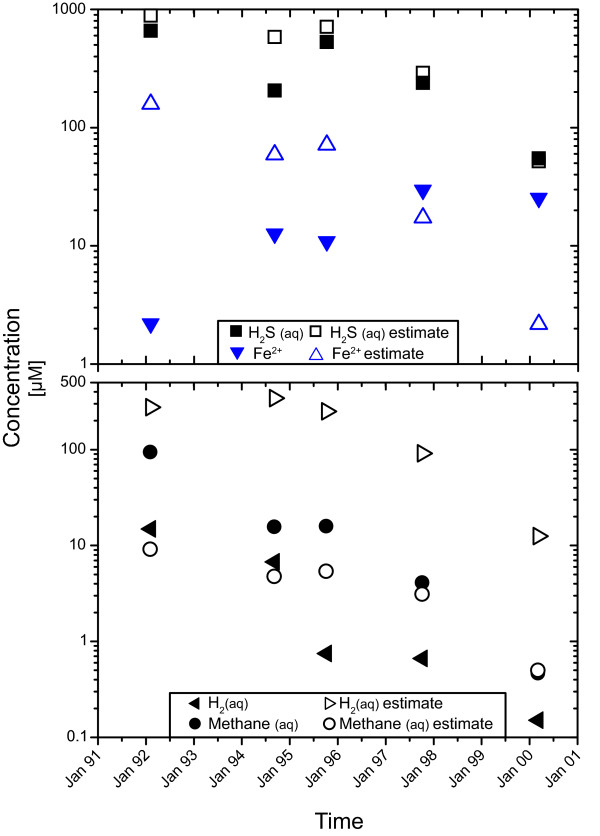
**Predicted and measured concentrations of iron, hydrogen sulfide, hydrogen, and methane for diffuse fluids in the Tube Worm Pillar area**. Hydrogen is strongly depleted over the entire period. Methane is enriched in the diffuse fluids, which may show methanogenesis in the subseafloor [13]. Until 1997, iron and H_2_S concentrations are generally lower than predicted from conservative mixing. In November of 1997 predicted and measured concentration are similar to each other. In April of 2000 measured H_2_S concentrations also correspond to the predictions from conservative mixing, but Fe-concentrations are higher than predicted in the diffuse fluid. Loss of Fe^2+ ^and H_2_S may be associated with precipitation of minerals. The surplus of measured Fe^2+ ^in 2000 could indicate hydrogenotrophic iron reduction.

The compositional changes of diffuse fluid compositions relative to the concentrations predicted from conservative mixing are depicted in Figure 4. Throughout the time series, H_2 _concentrations are decreased by 1.5 to 2 orders of magnitude relative to the concentrations expected from conservative mixing (cf. Table 1). Methane, in contrast, is enriched by a factor of ten relative to the value predicted from conservative mixing in February-March of 1992. In October 1994 and November 1995 this enrichment is about 3-fold. By 2000, measured methane corresponds to those predicted from conservative mixing, and no methane excess can be observed (Figure 4). The methane excess in 1992-1995 is consistent with the decrease in hydrogen, and ratios of H_2 _depletion to CH_4 _excess between 3 and 6 are consistent with the stoichiometry of the hydrogenotrophic methanogenesis reaction, from which that ratio would be predicted to be 4. In 1997 and 2000, however, methane excess was minimal and H_2 _depletion was still significant, suggesting that other hydrogen-consuming reactions may have also played a role.

While methane enrichment and depletion of hydrogen are indicators for methanogenesis, some of the methane may be metabolized shallower in the system prior to venting by aerobic or anaerobic respiration (Figure 1). There are indications from the Guaymas Basin that anaerobic oxidation of methane (AOM) may take place in vent settings and at temperatures > 30°C [32]. Our calculations suggest that AOM is energetically feasible, so a loss of methane through AOM may be possible. If this reaction took place in the subseafloor, depletions of methane should be associated with increased hydrogen sulfide concentrations. This trend is not observed. AOM may still be taking place, but the rates are too small to affect the compositions of the diffuse fluids.

**Figure 5:**
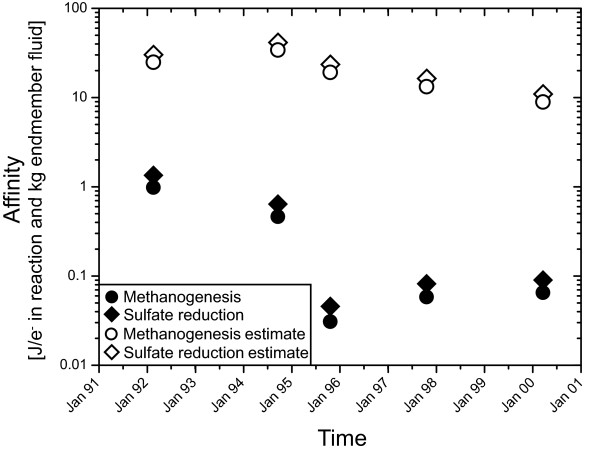
**Normalized affinities for sulfate reduction and methanogenesis in the Tube Worm Pillar area**. Affinities are high in the hypothetical fluids calculated from conservative mixing of seawater and hydrothermal fluid. In the measured fluids the affinity is strongly decreased. Notably, affinities drop markedly in the first three years, which reflects the decrease in H_2 _concentration in that time span (Figure 4). The removal of H_2 _and the lowering of affinity reflect the exploitation of H_2 _in fueling catabolic activity. During the last five years of the time series, normalized affinities had plateaued, possibly indicating a steady-state between hydrothermal energy supply and microbial utilization of energy.

Affinity calculations for hydrogenotrophic methanogenesis and sulfate reduction in the modeled fluid (Figure 5) indicate strong driving forces for both reactions. The affinity for the reaction is strongly controlled by the hydrogen activity, which has a power of 4 in the relevant mass action equations. Hydrogen endmember concentrations increase from 6460 μM in February/March of 1992 to 8910 μM in March of 1994. Hydrogen concentrations then decrease in the following years to 2700 μM in April of 2000 (Table 1). Unfortunately, the time series contains three points with data lacking for either the diffuse or endmember fluid. In March of 1994, the highest hydrogen concentration in the endmember fluid was measured, but no diffuse fluid was sampled. Hence, in our calculations, the sample collected in October of 1994 has the highest predicted hydrogen content (Figure 4) and also the highest normalized affinity for methanogenesis with 34.0 Joule per kg vent fluid and electron transferred in reaction (J/kg e^-^) or of 41.5 J/kg e^- ^for sulfate reduction in the predicted diffuse fluid (Figure 5). The fluids sampled in April of 2000 have the smallest fraction of vent fluid and the lowest hydrogen endmember concentration of 12.5 μM, yielding normalized affinities for methanogenesis of 8.9 J/kg e^- ^and 11.0 J/kg e^- ^for sulfate reduction. In these diffuse fluids, hydrogen concentrations are still lower than predicted from conservative mixing and they do not correlate with the endmember concentration or extent of mixing with seawater.

Overall, hydrogen concentrations decrease linearly within the first four years of the time series from 14.9 μM to 0.7 μM. Hydrogen concentrations then remain fairly constant in the range of 0.7 to 0.2 μM until 2000. Figure 5 shows the affinities of these reactions per mol electrons in the reaction and normalized to kg of vent fluid. The normalized affinities of sulfate reduction decrease from 1.4 J/kg e^- ^to 0.05 J/kg e^- ^in 1995 and rebounds to 0.08-0.09 J/kg e^- ^in the following years. Hydrogenotrophic methanogenesis has an affinity of 1.0 J/kg e^- ^in 1992 and decreases to 0.05 to 0.06 J/kg e^- ^in 1995 - 2000 (Table 2).

The differences in the affinities (Table 2) reflect the variability in hydrogen concentrations, but the magnitude of these differences is quite small, because the intensive term (Δ_r_G°) in the Gibbs energy calculation is very large for both reactions. The calculated affinities for methanogenesis and sulfate reduction (Table 2) lie above the estimated energy limit of microbial metabolism 10 kJ/8 e^- ^[33]. Minimum H_2 _concentrations required for microbial harnessing of hydrogen at the temperatures of diffuse venting (11.9 to 24.7°C) is on the order of 10^-8 ^M [33], which is considerably lower than the measured concentrations (> 2 × 10^-7 ^M). This result indicates that the microbial communities consume hydrogen but do not control its abundance. Apparently, hydrothermally driven influx of H_2 _into the system is overall greater than the rate at which H_2 _is metabolized. The constant hydrogen concentrations between 1995 and 2000 probably indicate some sort of steady-state between influx of hydrogen from below and hydrogen consumption in the subseafloor ecosystem. The early phase of decreasing hydrogen concentrations in the diffuse fluids is not related to changes in endmember compositions (steady between 250 and 350 μM), but may instead reflect the growth a hydrogenotrophic microbial community and increasing rates of consumption of H_2 _advected into the system by hydrothermal flow. In that early stage, there was a relation between H_2 _depletion and methane production, indicating that methanogenesis was responsible for both. In 1997 and 2000, H_2 _was still consumed, but the methane excess had disappeared. Instead, there was excess Fe in the fluids, suggesting that Fe-reduction was taking place, perhaps because it requires lower H_2 _activities than methanogenesis [33].

## Conclusions

Thermodynamic calculations of energy yields of catabolic reactions from geochemical data of diffuse fluids facilitate an assessment of microbial metabolism in vent settings. This has been demonstrated in two case studies, both from the EPR 9°50'N region, where published geochemical data [13,14] where used in systematic calculations of affinities of different catabolic reactions.

In the Bio9 area, affinities for sulfide oxidation strongly decrease, which is in accordance with the dying *Riftia *population. At the same time, an increase in the affinity for iron oxidation corresponds to a massive spread of red staining in the area, which is likely evidence for Fe-oxidizing bacteria. The results of the energy calculations verify the idea that the sudden change in vent fauna is a result of changes in fluid chemistry.

The example from the Bio9 area is more relevant to subseafloor processes. Enrichment of methane in diffuse fluids points to methanogenesis in the mixing and cooling zone. Our calculations confirm that hydrogenotrophic catabolic reactions have large energy yields throughout the duration of the time series (1992-2000). A large discrepancy in the amount of H_2 _predicted from conservative mixing and the measured H_2 _concentrations indicate effective scrubbing of H_2 _by subseafloor hydrogenotrophic microorganisms. During the first three years of the time series, affinities for hydrogenotrophic reactions decreased despite continued high H_2 _concentrations in the endmember fluids. This is interpreted to indicate the development of a hydrogenotrophic-based microbial ecosystem in the subseafloor. Between 1995 and 2000, the affinities remained constant and low (about an order of magnitude above the biological energy quantum). Apparently, influx of hydrogen from below and consumption of hydrogen within the subseafloor had reached a steady state. In 1997 and 2000, methane excesses were minimal, but the fluids showed pronounced enrichment of Fe relative to the concentrations predicted from conservative mixing. This finding may indicate a switch within the system from methanogenesis to Fe-reduction.

Our results show how thermodynamic calculations can be used to examine the relations between changes in fluid chemistry and seafloor biology. They are also a helpful tool in examining processes in the subseafloor and help highlight the tight relations and interdependencies between geochemistry and microbiology in vent systems.

## Competing interests

The authors declare that they have no competing interests.

## Authors' contributions

The study was developed jointly by both authors. MH conducted the thermodynamic calculations. Both authors were involved equally in the interpretations of the results and in writing and editing of the manuscript. All authors have read and approved the final manuscript.

## Supplementary Material

Additional file 1 **Table: Calculated normalized affinities for Aerobic sulfide oxidation and iron oxidation in J per e^- ^and kg Vent-fluid at the Northern Area (Bio9)**. Additional Data to Figure 3 Click here for file

## References

[B1] Jannasch HW Humphris SE, et al Microbial interactions with hydrothermal fluids Seafloor Hydrothermal Systems: Physical, Chemical, Biological, and Geological Interactions 1995 91 Washington, DC: American Geophysical Union, American Geophysical Union 273 273 Geophysical Monograph

[B2] McCollom TM Shock EL Geochemical constraints on chemolithoautotrophic metabolism by microorganisms in seafloor hydrothermal systems Geochimica et Cosmochimica Acta 1997 61 4375 4391 10.1016/S0016-7037(97)00241-X 11541662

[B3] Amend JP McCollom TM Hentscher M Bach W Catabolic and anabolic energy for chemolithoautotrophs in deep-sea hydrothermal systems hosted in different rock types Geochimica et Cosmochimica Acta 2011 75 5736 5748 10.1016/j.gca.2011.07.041

[B4] Shock E Holland ME Wilcock WSD, DeLong EF, Kelley DS, Baross JA, Cary SC Geochemical energy sources that support the subsurface biosphere The Subseafloor Biosphere at Mid-Ocean Ridges 2004 Washington, DC: American Geophysical Union 153 165 Geophysical Monograph

[B5] Houghton JL Seyfried WE Jr An experimental and theoretical approach to determining linkages between geochemical variability and microbial biodiversity in seafloor hydrothermal chimneys Geobiology 2010 8 457 470 10.1111/j.1472-4669.2010.00255.x 20726900

[B6] Huber JA Butterfield DA Baross JA Bacterial diversity in a subseafloor habitat following a deep-sea volcanic eruption FEMS Microbiology Ecology 2003 43 393 409 10.1111/j.1574-6941.2003.tb01080.x 19719671

[B7] Reysenbach A-L Liu Y Banta AB Beveridge TJ Kirshtein JD Schouten S Tivey MK Von Damm KL Voytek MA A ubiquitous thermoacidophilic archaeon from deep-sea hydrothermal vents Nature 2006 442 444 447 10.1038/nature04921 16871216

[B8] Kormas KA Tivey MK Von Damm K Teske A Bacterial and archaeal phylotypes associated with distinct mineralogical layers of a white smoker spire from a deep-sea hydrothermal vent site (9°N, East Pacific Rise) Environmental Microbiology 2006 8 909 920 10.1111/j.1462-2920.2005.00978.x 16623747

[B9] Perner M Bach W Hentscher M Koschinsky A Garbe-Schönberg D Streit WR Strauss H Short-term microbial and physico-chemical variability in low-temperature hydrothermal fluids near 5°S on the Mid-Atlantic Ridge Environmental Microbiology 2009 11 2526 2541 10.1111/j.1462-2920.2009.01978.x 19558512

[B10] Petersen JM Zielinski FU Pape T Seifert R Moraru C Amann R Hourdez S Girguis PR Wankel SD Barbe V Hydrogen is an energy source for hydrothermal vent symbioses Nature 2011 476 176 180 10.1038/nature10325 21833083

[B11] Amend JP Shock EL Energetics of overall metabolic reactions of thermophilic and hyperthermophilic Archaea and Bacteria FEMS Microbiology Reviews 2001 25 175 243 10.1111/j.1574-6976.2001.tb00576.x 11250035

[B12] Helgeson HC Mass transfer among minerals and hydrothermal solutions Geochemistry of hydrothermal ore deposits 1979 2 568 610

[B13] Von Damm KL Lilley MD Wilcock WSD, DeLong EF, Kelley DS, Baross JA, Cary SC Diffuse flow hydrothermal fluids from 9 50'N East Pacific Rise: evolution and biogeochemical controls The Subseafloor Biosphere at Mid-Ocean Ridges 2004 Washington, DC: American Geophysical Union 245 268 Geophysical Monograph

[B14] Von Damm KL German CR, Lin J, Parson LM Evolution of the hydrothermal system at East Pacific Rise 9 50'N: Geochemical evidence for changes in the upper oceanic crust Mid-Ocean Ridges: Hydrothermal Interactions between the Lithosphere and Oceans 2004 Washington, DC: American Geophysical Union 285 304 Geophysical Monograph

[B15] Carroll S Mroczek E Alai M Ebert M Amorphous silica precipitation (60 to 120°C): comparison of laboratory and field rates Geochimica et Cosmochimica Acta 1998 62 1379 1396 10.1016/S0016-7037(98)00052-0

[B16] Edmond JM Measures C McDuff RE Chan LH Collier R Grant B Gordon LI Corliss JB Ridge crest hydrothermal activity and the balances of the major and minor elements in the ocean: The Galapagos data Earth and Planetary Science Letters 1979 46 1 18 10.1016/0012-821X(79)90061-X

[B17] Bethke C Geochemical reaction modeling: Concepts and applications 1996 Oxford University Press, USA

[B18] Johnson JW Oelkers EH Helgeson HC SUPCRT92: A software package for calculating the standard molal thermodynamic properties of minerals, gases, aqueous species, and reactions from 1 to 5000 bar and 0 to 1000°C Computers & Geosciences 1992 18 899 947 10.1016/0098-3004(92)90029-Q

[B19] Dick J Calculation of the relative metastabilities of proteins using the CHNOSZ software package Geochemical Transactions 2008 9 18834534 10.1186/1467-4866-9-10 PMC2654789

[B20] Wolery T Jove-Colon C US Department of Energy Qualification of thermodynamic data for geochemical modeling of mineral-water interactions in dilute systems Energy 2004 Bechtel SAIC Company, Las Vegas, Nevadam, USA

[B21] Drummond SE Boiling and mixing of hydrothermal fluids Ph D thesis 1981 University Microfilms Int.

[B22] Tivey MK Humphris SE Thompson G Hannington MD Rona PA Deducing patterns of fluid flow and mixing within the TAG active hydrothermal mound using mineralogical and geochemical data J Geophys Res 1995 100 12527 12555 10.1029/95JB00610

[B23] Foustoukos DI Houghton JL Seyfried WE Jr Sievert SM Cody GD Kinetics of H_2_-O_2_-H_2_O redox equilibria and formation of metastable H_2_O_2 _under low temperature hydrothermal conditions Geochimica et Cosmochimica Acta 2011 75 1594 1607 10.1016/j.gca.2010.12.020

[B24] McCollom TM Geochemical constraints on sources of metabolic energy for chemolithoautotrophy in ultramafic-hosted deep-sea hydrothermal systems Astrobiology 2007 7 933 950 10.1089/ast.2006.0119 18163871

[B25] Fornari DJ Shank T Von Damm KL Gregg TKP Lilley M Levai G Bray A Haymon RM Perfit MR Lutz R Time-series temperature measurements at high-temperature hydrothermal vents, East Pacific Rise 9°49'-51'N: evidence for monitoring a crustal cracking event Earth and Planetary Science Letters 1998 160 419 431 10.1016/S0012-821X(98)00101-0

[B26] Sohn RA Fornari DJ Von Damm KL Hildebrand JA Webb SC Seismic and hydrothermal evidence for a cracking event on the East Pacific Rise crest at 9° 50' N Nature 1998 396 159 161 10.1038/24146

[B27] Shank TM Fornari DJ Von Damm KL Lilley MD Haymon RM Lutz RA Temporal and spatial patterns of biological community development at nascent deep-sea hydrothermal vents (9°50'N, East Pacific Rise) Deep Sea Research Part II: Topical Studies in Oceanography 1998 45 465 515 10.1016/S0967-0645(97)00089-1

[B28] Cavanaugh CM Symbiotic chemoautotrophic bacteria in marine invertebrates from sulphide-rich habitats Nature 1983 302 58 61 10.1038/302058a0

[B29] Toner BM Santelli CM Marcus MA Wirth R Chan CS McCollom T Bach W Edwards KJ Biogenic iron oxyhydroxide formation at mid-ocean ridge hydrothermal vents: Juan de Fuca Ridge Geochimica et Cosmochimica Acta 2009 73 388 403 10.1016/j.gca.2008.09.035

[B30] Betts JN Holland HD The oxygen content of ocean bottom waters, the burial efficiency of organic carbon, and the regulation of atmospheric oxygen Palaeogeography, Palaeoclimatology, Palaeoecology 1991 97 5 18 10.1016/0031-0182(91)90178-T 11538093

[B31] Proskurowski G Lilley MD Olson EJ Stable isotopic evidence in support of active microbial methane cycling in low-temperature diffuse flow vents at 9°50'N East Pacific Rise Geochimica et Cosmochimica Acta 2008 72 2005 2023 10.1016/j.gca.2008.01.025

[B32] Schouten S Wakeham SG Hopmans EC Sinninghe Damste JS Biogeochemical evidence that thermophilic archaea mediate the anaerobic oxidation of methane Appl Environ Microbiol 2003 69 1680 1686 10.1128/AEM.69.3.1680-1686.2003 12620859 PMC150050

[B33] Hoehler TM Biological energy requirements as quantitative boundary conditions for life in the subsurface Geobiology 2004 2 205 215 10.1111/j.1472-4677.2004.00033.x

